# Understanding acute ankle ligamentous sprain injury in sports

**DOI:** 10.1186/1758-2555-1-14

**Published:** 2009-07-30

**Authors:** Daniel TP Fong, Yue-Yan Chan, Kam-Ming Mok, Patrick SH Yung, Kai-Ming Chan

**Affiliations:** 1 Department of Orthopaedics and Traumatology, Prince of Wales Hospital, Faculty of Medicine, The Chinese University of Hong Kong, Hong Kong, PR China; 2 The Hong Kong Jockey Club Sports Medicine and Health Sciences Centre, Faculty of Medicine, The Chinese University of Hong Kong, Hong Kong, PR China; 3 Department of Orthopaedics and Traumatology, Alice Ho Miu Ling Nethersole Hospital, Hong Kong, PR China

## Abstract

This paper summarizes the current understanding on acute ankle sprain injury, which is the most common acute sport trauma, accounting for about 14% of all sport-related injuries. Among, 80% are ligamentous sprains caused by explosive inversion or supination. The injury motion often happens at the subtalar joint and tears the anterior talofibular ligament (ATFL) which possesses the lowest ultimate load among the lateral ligaments at the ankle. For extrinsic risk factors to ankle sprain injury, prescribing orthosis decreases the risk while increased exercise intensity in soccer raises the risk. For intrinsic factors, a foot size with increased width, an increased ankle eversion to inversion strength, plantarflexion strength and ratio between dorsiflexion and plantarflexion strength, and limb dominance could increase the ankle sprain injury risk. Players with a previous sprain history, players wearing shoes with air cells, players who do not stretch before exercising, players with inferior single leg balance, and overweight players are 4.9, 4.3, 2.6, 2.4 and 3.9 times more likely to sustain an ankle sprain injury. The aetiology of most ankle sprain injuries is incorrect foot positioning at landing – a medially-deviated vertical ground reaction force causes an explosive supination or inversion moment at the subtalar joint in a short time (about 50 ms). Another aetiology is the delayed reaction time of the peroneal muscles at the lateral aspect of the ankle (60–90 ms). The failure supination or inversion torque is about 41–45 Nm to cause ligamentous rupture in simulated spraining tests on cadaver. A previous case report revealed that the ankle joint reached 48 degrees inversion and 10 degrees internal rotation during an accidental grade I ankle ligamentous sprain injury during a dynamic cutting trial in laboratory. Diagnosis techniques and grading systems vary, but the management of ankle ligamentous sprain injury is mainly conservative. Immobilization should not be used as it results in joint stiffness, muscle atrophy and loss of proprioception. Traditional Chinese medicine such as herbs, massage and acupuncture were well applied in China in managing sports injuries, and was reported to be effective in relieving pain, reducing swelling and edema, and restoring normal ankle function. Finally, the best practice of sports medicine would be to prevent the injury. Different previous approaches, including designing prophylactice devices, introducing functional interventions, as well as change of games rules were highlighted. This paper allows the readers to catch up with the previous researches on ankle sprain injury, and facilitate the future research idea on sport-related ankle sprain injury.

## Review

### Introduction

Ankle ligamentous sprain injury is the most common single type of acute sport trauma [1]. Over the years, various preventive strategies have been implemented, however, a recent epidemiology revealed that ankle sprain injury still dominated in sport injury, as it accounted for 14% of all attendance in an accident and emergency department [2]. In the recent decade, the growing orthopaedic biomechanics techniques have enhanced a better understanding of injury mechanism, and the subsequent research in sports injury prevention and management [3]. This paper summarizes the current understanding in acute ankle ligementous sprain injury in sports, which facilitates the future research on ankle sprain prevention. Literature search of MEDLINE (from 1966) and PubMed (from 1950) was conducted in May 2008. The search keyword string was "ankle AND (injury OR injuries OR sprain)", which appeared in the title, abstract or keyword fields. The title and abstract of each entry was read to identify and exclude unrelated articles. Articles not written in English were also excluded. The information of the papers was summarized into the sub-topics in this article in the following paragraphs to form the current understanding on acute ankle ligamentous sprain injury in sports.

### Sports participation and sports injury

All around the world, medical doctors and sports scientists were actively promoting regular physical exercises to gain health benefits and to prevent cardiovascular related disease [4]. People nowadays are more eager in participating in sports and exercises for personal interest, leisure, relaxation, health and fitness purposes. In Hong Kong, according to the annual survey of sports participation conducted by the Hong Kong Sports Institute [5], people in general were becoming more active in sports participation from 1996 to 2001. The increasing trend was found in youngster and elderly [6] as well as working population [7]. The increasing sports participation was also reflected by the number of participants in the annual marathon race. There were only 1,000 participants in the first marathon race in 1997. The number of participants increased every year, and reached 10,000 in 2001. The number of participants kept increasing in recent years and has dramatically increased to 50,000 in 2008 [8]. Most of the participants were recreational athletes, indicating a mass participation of sports among the population.

However, in contrary to the promotion of the health benefits from sports participation, sports often cause injuries [9]. A study in Sweden [10] reported that 17% of the 3,341 acute visits to a clinic due to accidents in a one-year prospective study were from sports. It was comparable to home accident (26%), work accident (19%) and was much higher than traffic accident (7%). In United Kingdom, there were 7.1% of the 2,432 new patients attending accident and emergency department in a 10-day period sustained trauma from sports [11]. In North Ireland, for adolescent of age 11–18 who actively participated in sports, as much as 51% of the attendees sustained sports injuries [12]. When the sports participation rate became high, the exposure to potential injury increased and thus the high incidence of sport injury [13].

### Problem and outcome of sports injury

Sports injuries resulted in pain [14], loss of playing or working time [15], as well as medical expenditure [16]. Severe ankle injuries also occasionally resulted in bone fractures [17], functional instability [18], decreased muscle strength [19], inferior proprioception [20], limited mobility [21], disability [22], permanent cease or retirement of sports participation [23]. Without adequate treatment and rehabilitation, sports injuries may also cause significant susceptibility in developing osteoarthritis [24] and other kinds of permanent sequalae [25]. For world-class and commercial sports teams, absence of key players due to unexpected injuries may result in defeats in major games and huge economic loss.

### Prevalence and patterns of ankle sprain injury

Previous studies reported that injury to the knee was the most common and injury to the ankle the next [9,26]. Ankle was the most common injured body site in 24 of 70 included sports [1]. Ankle ligament sprain were also reported to be the most common injury for college athletics in the United States [27]. In Hong Kong, a survey on 2,293 patients attending a sports injury clinic reported that the knee (27.3–50.5%) and the ankle (16.8–24.7%) were the most common sites of injury in soccer, basketball, volleyball and long-distance running sports [9]. In marathon racing, another study on 580 runners in a marathon race reported that 33.9% of the injuries were to the knee and 20.9% were to the ankle [28]. Among ankle injuries, ankle sprain injury accounts for more than 80%, and is also the most common single type of sport-related trauma among all body sites and types [29-31]. Among ankle sprain injuries, 77% were lateral sprains [22] and 73% involved isolated rupture or tear to the anterior talofibular ligament [32,33]. A local survey conducted on 380 athletes with 563 sprained ankles reported that the majority of these injured athletes were pursuing running and jogging activities (25%), racquet sports (20%), ball games (19%) and soccer (14%) [34]. The residual problem included pain (30.2%), instability (20.4%), crepitus (18.3%), weakness (16.5%), stiffness (14.6%) and swelling (13.9%).

### Ankle anatomy and biomechanics

In human anatomy, the ankle joint is where the foot and the leg segments meet. It comprises of three major articulations: the talocrural joint, the subtalar joint, and the distal tibiofibular syndesmosis [18]. The talocrural joint is also termed the tibiotalar joint or the mortise joint, and is formed by the articulation of the dome of talus, the tibial plafond, the medial malleolus and the lateral malleolus. This joint, in isolation, behaves rather like a hinge joint that allows mainly plantarflexion and dorsiflexion. The fibula extends further to the lateral malleous than the tibia does to the medial malleolus, thus creating a block to eversion [35]. Such body feature mainly allows larger range of inversion than eversion, thus, inversion sprains are more common than eversion ones [36].

The talocrural joint is supported by several main ligaments, namely the anterior talofibular ligament (ATFL), the calcaneofibular ligament (CFL) and the posterior talofibular ligament (PTFL) at the lateral aspect, and the deltoid ligament in the medial aspect of the ankle [37]. Among the lateral ligaments, the ATFL is the weakest as it has the lowest ultimate load, approximately 138.9N, which is about half of that of PTFL, that is, 261.2N, and one-third of that of CFL, that is, 345.7N [38]. These values were obtained from mechanical test on ligaments of fresh human ankles. ATFL is approximately 20–25 mm long, 7–10 mm wide and 2 mm thick [39,40]. It originates from the anterior-inferior border of the fibular and inserts to the neck of the talus [30]. It prevents anterior displacement and internal rotation of the talus, especially when the talocrural joint is plantarflexed [41-43]. Due to its low ultimate load and the anatomical positions of origins and insertions, the ATFL is most commonly injured in a lateral ankle sprain [30].

The subtalar joint is formed by the articulation between the bottom of the talus and the calcaneus [18]. It consists of two separate joint cavities. First, the anterior subtalar joint, or also termed the talocalcaneonavicular joint, is formed from the head of the talus, the anterior-superior facets, the sustentaculum tali of the calcaneus, and the concave proximal surface of the tarsal navicular [44]. Second, the posterior subtalar joint is formed between the inferior posterior facet of the talus and the superior posterior facet of the calcaneus [45]. The anterior and posterior subtalar joints behave like a single ball-and-socket joint and share a common oblique axis of rotation [46], which averages a 42-degree upward tilt and a 23-degree medial angulation from the perpendicular axes of the foot [47]. This articulation allows inversion and eversion, or supination and pronation as described as a triplanar motion [36]. The subtalar joint is supported by three groups of ligaments, namely the deep ligaments, the peripheral ligaments, and the retinacula [48]. Together these ligament groups stabilize the subtalar joint and form a barrier between the anterior and posterior joint capsules [18].

The distal tibiofibular syndesmosis is formed by the articulation between the distal tibia and fibula [49]. The joint is mainly stabilized by a thick interosseous membrane, with the anterior and posterior inferior tibiofibular ligaments, to form the stable roof for the mortise of the talocrural joint [18]. This joint allows limited translation and rotation during talocrural dorsiflexion and plantarflexion to accommodate the asymmetric talus while maintaining congruency [50]. Injury to this ligament group is rare, and is often termed ankle syndesmosis injury [49], syndesmotic ankle sprain [51], or high ankle sprain [52].

### Risk factors for ankle sprain injury

Risk factors were commonly classified as extrinsic or intrinsic [53]. Extrinsic risk factors are those that come from outside of the body, while intrinsic factors are those from within the body. In 1997, Barker, Beynnon and Renstrom [54] did a comprehensive review on the ankle injury risk factors in sports as reported by about 20 prospective studies. For extrinsic factors, although they found some discrepancies among the included studies, they generally reported that the prescription of orthosis, but not high-top shoes, could help decreasing the risk of sustaining ankle sprain injury in players with previous sprain history. Increased exercise intensity in soccer raised the injury risk, but the player positions in soccer and basketball did not cause any difference. For intrinsic factors, they reported that a previous sprain history, a foot size with increased width, an increased ankle eversion to inversion strength, plantarflexion strength and ratio between dorsiflexion and plantarflexion strength, and limb dominance could increase the ankle sprain injury risk. The foot type, indication of ankle instability, and high general joint laxity were identified not to be risk factors. In 2002, Beynnon, Murphy and Alosa [55] conducted another comprehensive literature review and reported a consensus that gender, general joint laxity and foot type were not risk factors for ankle sprain injury. In 2007, Morrison and Kaminski [56] suggested that the cavovarus deformity, increased foot width, and increased calcaneal eversion range of motion were related to the occurrence of lateral ankle sprain injury. However, significant discrepancies were found with regard to whether or not height, weight, limb dominance, ankle joint laxity, anatomical alignment, muscle strength, muscle reaction time, and postural sway are risk factors for ankle sprain injury.

Some recent studies reported that players with a history of ankle sprain, players wearing shoes with air cells in the heel, and players who did not stretch before exercising were 4.9, 4.3 and 2.6 times more likely to sustain an ankle sprain injury [57]. People with inferior single leg balance [58] and overweight [59] were 2.4 and 3.9 times more likely to have sprain injury respectively. Reduced ankle dorsiflexion range [60], the use of artificial turf for soccer [61] and having a posteriorly positioned fibula [62] were also reported as risk factors. In 2005, Willems and coworkers [63] investigated some dynamic risk factors during gait in related to ankle sprain injury. They reported that for subjects who were at risk of sustaining an inversion sprain, a laterally situated center of plantar pressure was found at initial contact during the stance phase. The same research group also reported the intrinsic risk factors for inversion ankle sprain for male and female. For male, a slower running speed, less cardiorespiratory endurance, less balance ability, decreased dorsiflexion muscle strength, decreased dorsiflexion range of motion, less coordination ability, and faster reaction of the tibialis anterior and gastrocnemius muscles were the significant risk factors [64]. For female, a less accurate passive joint inversion position sense, a higher extension range of motion at the first metatarsophalangeal joint, and a less coordination of postural control were the major risk factors [65]. However, we have to be aware that these risk factors are only some correlations with ankle ligament sprain injury. They may not be the direct cause, or the aetiology of ankle ligament sprain.

### Aetiology of ankle supination sprain injury

Fuller [66] suggested that most ankle sprain injuries were caused by an increased supination moment at subtalar joint, which was often a result of the position and the magnitude of the vertically projected ground reaction force at initial foot contact. If the center of plantar pressure deviated medially to the subtalar joint axis, a greater moment arm along the subtalar joint axis was achieved and thus the subsequent increased supination moment to initiate sudden explosive ankle supination. Wright and coworkers [67] conducted a computational forward dynamic simulation study and reported that increased touch down plantarflexion caused increased ankle sprain occurrences. When a foot was plantarflexed during touch down, the contact to the ground was made with the forefoot, thus increased the moment arm among the subtalar joint axis and also the resultant joint torque to cause sudden explosive twisting motion and ankle sprain injury. Therefore, foot positioning during touch down was identified as an aetiology of ankle sprain injury. This also supported the suggestion that ankle taping or bracing corrected ankle joint positioning at landing rather than provided mechanical support to the ankle joint [68-70].

Another aetiology of ankle sprain injury is the delayed reaction time of the peroneal muscles at the lateral aspect of the ankle. Ashton-Miller and coworkers [69] suggested that an ankle sprain injury occured in 40 milliseconds (ms), as the vertical ground reaction force peaked at about 40 ms when landing from a jump [69]. At the lateral aspect of the human ankle, the peroneal muscles, including the peroneal longus and peroneal brevis, function to initiate ankle pronation which opposes the ankle supination motion [70]. Numerous research groups reported the reaction time of the peroneal muscles to be 50 ms or more. For instance, in sudden inversion tests with healthy subjects in an initial standing position, the peroneal muscle reaction time was reported to be 57–58 ms [71], 57–60 ms [72], 58 ms [73], 65–69 ms [74], 67–69 ms [75] and 69 ms [76]. For patients with ankle instability, the peroneal reaction time is longer – it was reported to be 82–84 ms [77] and 85 ms [78]. In a sudden inversion in a dynamic walking trial, the reaction time is also longer, as reported to be about 74 ms [72]. After the reflex response, the eversion torque was generated at 135 ms [79] and the subsequent active eversion was achieved at about 176 ms [80]. Therefore, it is postulated that the human reflex response is not fast enough to accommodate the sudden explosive motion in a sprain injury.

### Mechanism and biomechanics of ankle supination sprain injury

Understanding the injury mechanism is very important for the research of injury prevention [81,82]. In ankle supination sprain, there is ankle inversion plus an internal twisting of the foot [83], and plantarflexion with the subtalar joint adducting and inverting [84]. Sometimes there is also an external rotation of the lower leg in respect to the ankle joint [85]. Stormont and coworkers [86] suggested that most ankle sprains occurred during systematic loading and unloading, but not while the ankle was fully loaded due to articular restraints. When the foot is in plantarflexion, the anterior talofibular ligament is often injured; when the foot is in dorsiflexion, the calcaneofibular ligament is often injured [39]. In soccer, most ankle sprains were sustained during player contact (59%), but during non-contact situations for goalkeepers (79%) [32]. In a recent study to analyze the ankle supination sprain injury with video, Andersen and coworkers [87] reported that there were two major mechanisms: (1) impact by opponent on the medial aspect of the leg just before or at foot strike, resulting in a laterally directed force causing the player to land with the ankle in a vulnerable inverted position; (2) forced plantar flexion when the injured player hit the opponent's foot when attempting to shoot or clear the ball. Most of these mechanisms finally led to the rupture of the anterior talofibular ligament, as this ligament often sustained higher strain and strain rate values than the other ligaments at the lateral ankle [88].

The biomechanics of ankle supination sprain injury was seldom reported in the literature, as it is practically impossible and also unethical to conduct systematic dynamic ankle sprain test in the laboratory. Previous mechanism study only reported qualitative information. For quantitative evaluation, different research groups managed to conduct cadaver study to understand the ankle biomechanics during simulated spraining tests. In static cadaver study, Markolf, Schmalzried and Ferkel reported that a 41–45 Nm external rotatory torque would cause ankle failure [89], as defined by a major drop-off of the torque as the foot continued to rotate, indicating a bony fracture or ligamentous rupture. In dynamic cadaver study, Self, Harris and Greenwald [88] studied the ankle biomechanics during a drop test with cadaver ankles, to simulate the scenario in landing technique in sports [90]. For systematically evaluation, Wright and coworkers [67,91] conducted a computational forward dynamic simulation study to investigate the ankle biomechanics during landing on irregular surfaces. Yet there was only one quantitative investigation on the ankle biomechanics during a real ankle sprain injury scenario being reported [92]. An accidental supination sprain injury was analyzed, where the ankle sprain injury occurred in a laboratory under a high-speed video capturing setting. It reported that the ankle joint reached an inversion of 48 degrees and an internal rotation of 10 degrees.

### Diagnosis of acute ankle sprain injury

It is not uncommon for primary care physicians to misdiagnose various ankle problems as simple ankle sprains [93], and thus it is important to have a good differential diagnosis system for every acute ankle sprain injury (Table 1). Lynam [94] presented a protocol for the nurse to assess acute foot and ankle sprain at the emergency room, while Harmon [36] presented a systematic approach that consists of five steps to avoid missing potentially serious injuries: (1) palpation of bony structures, (2) palpation of ligamentous structures, (3) assessment of range of motion of the ankle, (4) testing of ankle muscles, and (5) special tests. Firstly, the diagnosis of fracture injuries was important as these patients normally have to be admitted to wards for emergency operative treatments [95]. Ankle fracture injuries were commonly diagnosed with the use of radiography [96], or the Ottawa Ankle Rules [97] which had a nearly 100% sensitivity [98,99] – it could significantly reduce the routine use of radiography [100,101]. Secondly, palpating ligamentous structures gave an idea that which ligament is probably injured. This can also be done together with the range of motion test, especially in voluntary dorsiflexion and plantaflexion. By doing the range of motion test, the physician could at the same time examine the ankle muscles.

**Table 1:** Summary of assessments for ankle injury

Testes	Descriptions (Sensitivity and specificity included if data is available)
Lynam [94]	An examination to assess acute foot and ankle sprain at emergency room.
Harmon [95]	A systematic approach that consists of five steps to avoid missing potentially serious injuries.
Ottawa ankle rules [97]	Rules to decide whether patients with acute ankle injury need X-ray or radiography. 100% sensitivity and 40% specificity for detection of malleolus fractures.
Radiography [100,101,107]	Stress radiography was performed with manual maximum force in inversion. The talar tilt was measured as the angle between the horizontal skeletal joint surfaces of the talus and the tibia.
Anterior drawer test [40,102]	Assesses the stability of the ATFL by cupping the heel in one hand and pulling it forward while stabilizing the tibia with other hand.
Talar tilt test [102]	Both ATFL and CFL were accessed, while ankle is inverted and the laxity was compared with that of the uninjured side.
Eversion stress test [103]	Heel was gently grasped with one hand, and the tibia with the other hand. A varus and then a valgus tilt stress were applied to the heel.
External rotation test [50]	Knee and ankle at 90 degree and a force with external rotation is applied to the midfoot area. Test is positive with pain.
Magnetic resonance imaging [99,104]	5-point grading system using noninvasive, high-resolution MRI to evaluate the articular cartilage of the talar dome. Sensitivity is 39% and Specificity is 50% for diagnose ankle-ligamentous injury.
Arthography [105]	After passive manipulation of the foot for 2 min, radiographs were obtained in 20 degrees internal rotation, anteroposterior projection, 40 degrees external rotation, lateral projection, and a soft tissue view just caudal to the lateral malleolus. Sensitivity is 100%.
Sonography [106]	Patient lay on the side of unaffected leg with the knee joint flexed to 90 degrees, while the affected leg was only slightly flexed. Sensitivity is 92% and specificity is 83%.
3D computed tomography [107]	3D CT images were obtained with a multidirector CT scanner. The patient was placed in supine position with the neutral position of bilateral ankle joint. Accuracy to diagnose ATFL tears is 94.4%.

When fracture is ruled out, specific special tests should be performed in order to correctly diagnose if the problem is a ligamentous injury. The anterior drawer test and the talar tilt test were the two common tests to assess the integrity of the anterior talofibular ligament, and could be useful in diagnosing the grading of the tear of the ligament [39,102]. To test the medial ligament, mainly the deltoid ligament at the medial aspect of the ankle, the eversion stress test was commonly performed [103]. This could be tested by the external rotation test and the squeeze test [49]. Sometimes these specific tests are performed together with radiography, that is, the stress radiography test [96]. There were also some other devices and techniques to assist the diagnosis: magnetic resonance imaging [104], arthrography [105], sonography [106], three-dimensional computed tomography [107], bone scintography [99], and arthroscopic diagnosis [108].

Beside ligamentous injuries, the problem could be a tendon rupture. The rupture of the Achilles tendon at the rear ankle could be examined by Thompson test [109], where the calf of the patient was squeezed with knee flexed. If the foot moved with the plantar flexion maneuver, the Achilles tendon was at least partially intact. Peroneal tendon rupture was less sensitive to the physical examination such as subluxation test and stress radiographic assessment, and may sometimes require tendonscopy [110] or surgical exploration [111,112] to confirm the diagnosis.

### Grading systems for evaluating acute ankle ligamentous sprain

There were numerous grading systems to grade an acute ankle ligamentous sprain injury [113], as summarized in Table 2. The two most basic systems were the Anatomic System which grades the injury in three grades accordingly to the ligaments that have been damaged, and the American Medical Association Standard Nomenclature System which considers the severity of the injury to the ligaments [114]. There were also some three-grade systems that grade the injury according to combined clinical presentation from the anatomical damage, severity of injury, and associated injuries of the surrounding structures [115-117]. Davis and Trevino [118] presented a staging system which consisted of four grades with some sub-grading to grade an ankle injury accordingly to the pathology, that was the damage to the ligamentous structure, and also the instability as presented clinically. Mann and coworkers [113] devised a practical system for outpatient clinical use. It was based on three items – pain, swelling and inability to walk. Each item was rated with 0 to 3 points (0 = none, 1 = mild, 2 = moderate, 3 = severe), and a total score was summed up for the final grading: Grade I: 1–3 points, Grade II: 4–6 points, Grade III: 7–9 points.

**Table 2:** Summary of grading scales to classify ankle sprain injury.

Grading scale	Description	Grading	Static/dynamic
American Medical Association Standard Nomenclature System [114]	Considers the severity of the injury to the ligaments.	-	Static
Davis and Trevino [118]	Grading according to pathology, ie the damage to the ligamentous structure and also the instability presented clinincally.	Fours grades with some sub-grading.	Static
Mann [113]	Grading according to swelling, sensitivity to pressure, drawer test, tilt test, ability of jumping, running, cutting.	Each of the three item is rated with 0–3 points (0 = none, 1 = mild, 2 = moderate, 3 = severe), a total score for final grading: Grade I: 1–3 points, Grade II: 4–6 points, Grade III 7–9 points.	Static
Jaikkomen, Kannus and Jarvinen [119]	Three questions on subjective assessment, two clinical measurements on the ankle, two muscle strength tests, one ankle functional stability test and one balancing test.	Four classes grading system. Score for fully normal ankle was 100. the total score of 85 to 100 was graded as excellent, 70–80 as good, 55–65 as fair and > = 50 as poor.	Dynamic
De Bie el. Al. [120]	Evaluates the pain, instability, weight bearing, swelling and gait pattern and adds up to a score of 100.	Cutoff point for being healed was defined as obtaining more than 75 points on a function score and scoring less than two out of 12 points on the palpation or stress test. To score as being able to walk, minimal 35 points need to be obtained.	Dynamic
Clanton [114]	Relates to the treatment protocols requested.	Stable or unstable ankle (subgrade of non-athletics, older patients and young active athletes).	Dynamic

For dynamic functional evaluation, Kaikkonen, Kannus and Jarvinen [119] devised a performance test protocol with scoring scale, which consisted of three questions on subjective assessment, two clinical measurements on the ankle, two muscle strength tests, one ankle functional stability test and one balancing test, for evaluating ankle injuries. The total score correlated very well with the isokinetic strength test of the ankle, the subjective opinion about the recovery, and also the subjective function assessment, and thus the protocol was practical for clinical evaluation of ankle sprain injury. de Bie and coworkers [120] derived an ankle functional scoring system which evaluated the pain, instability, weight bearing, swelling and gait pattern and added up to a score of 100. Clanton [114] devised another system that related to the treatment protocols requested. The system consisted of two main classes which categorized the injured ankle as a stable or unstable one. The stable group was suggested to receive symptomatic treatment for pain relief. For the unstable category, another sub-category classified the patient to be non-athletes or older patients, and young active athletes. Non-athletes and older patients were suggested to receive functional treatment. For the young active athletes group, there was one more layer to divide the patients to be with negative stress radiograph findings, with positive tibio-talar stress radiograph findings, and with subtalar instability. Those with positive tibio-talar instability were suggested to consider operative surgical repair of the ligament complex.

### Management of acute ankle ligamentous sprain

When an acute ankle ligamentous sprain happened, it was often the team physician to give immediate on-field care to the injured athletes [121]. The aim was to remove the injured athlete from the field and protect the athlete from further injury, instead of to diagnose the injury immediately, as the accuracy of an on-field diagnosis was reported to be inadequate [122]. Table 3 shows the details of some common treatments. Most commonly, the RICE (Rest, Ice-cooling, Compression, Elevation) treatment was delivered to the patients both on-field [123] and at the accident and emergency department [124,125]. Cryotherapy, or ice-cooling, may help in reducing pain in the first week after the injury [126], however, compression and elevation could only decrease ankle swelling temporarily and the effect lasted for less than five minutes after the limb was returned to a gravity-dependent position [127], and may even lead to discomfort and the need for analgesia after the application of the double Tubigrip compression bandage [128].

**Table 3:** Summary of the treatment Methods for ankle sprain injury.

Treatment	Effect	Detail
Aircast ankle brace [136]	Significant improvement in ankle joint function at both 10 days and one month compared with standard management with an elastic support bandage.	Application of a semi-rigid ankle brace consists of two contured thermoplastic lateral straps lined with foam pads and designed to fit against the medial and lateral malleoli of the ankle joint. The aircells can be supplemented with additional air through an inlet port. The rigid sidewalls are held in place with Velcro strapping.
Elastic support bandage [136]	Inprove single-leg-stance balance and might decrease the likelihood of future sprains.	-
Training on wobble board [137]	Anteroposterior and mediolateral stability improved after training.	Patient practices balancing on a rectangular or square platform with a single plane-rounded fulcrum underneath that extends the width of the board.
Ankle disk training [138,139]	Balanced improved after training.	Patients have to balance the circular platform with hemispherical ball underneath, without allowing the edges of the platform to touch the floor.
Imagery [141]	Greater muscle endurance than the control group.	Movement imagery, including visual imagery of movement itself and imagery of kinaesthetic sensations.
Resistive walking boot [146]	-	Patients' ankle were immobilize by walking boot with aircast support and compression wrap in the first 0–5 days after injury.
Traditional Chinese medicine methods [141,147-150]	-	Drug treatment, electroacupuncture, massages.

In five days after the initial injury, the sensitivity of physical examination to ankle injury improved gradually after the pain and swelling had diminished [129]. Appropriate treatments could be delivered accordingly to the diagnosis of the injured ankle. There was a general consensus to conservatively treat grade I and II ankle ligamentous injuries with functional exercises [130-132]. Trevino, Davis and Hecht [133] and Mattacola and Dwyer [134] presented some functional treatment protocols to manage ankle ligament injuries, which consisted of various modalities such as flexibility exercises, strength and balancing training, ankle joint proprioception and muscular strength training, isometric and isotonic strength training, and even exercises in water. Kerkhoffs and coworkers [135] conducted a systematic review and reported that functional exercises were more effective than immobilization in terms of return to sports, return to work, reduce persistent swelling, restore ankle stability, restore range of motion, and patient satisfaction.

The effect of other conservative treatments was reported by different research groups. Boyce, Quigley and Campbell [136] reported that the use of an Aircast ankle brace produced significant improvement in ankle joint function in 10 days and one month compared with an elastic support bandage. Madras and Barr [137] reported that ankle disk training on wobble board were effective in enhancing single leg balance and reducing recurrent sprain injury, while Osborne and coworkers [138] and Sheth and coworkers [139] reported the effect of ankle disk training in enhancing peroneal muscle reaction time. De Simoni and coworkers [140] suggested that a 12-week prescription of orthosis was effective in improving functional stability at the ankle joint. Recently, Christakou, Zervas and Lavallee [141] suggested that imagery may be effective in improving muscle endurance in the rehabilitation of grade II ankle sprain.

For grade III ankle sprain, the treatment was still controversial [131]. Some surgeons recommended surgical repair [142], and some favored non-operative conservative treatment [143]. In 2000, Pijnenburg and coworkers [144] conducted a meta-analysis and suggested that primary operative repair of lateral ankle ligaments led to better results concerning recurrent giving way and pain on activity when compared with conservative treatment. However, in 2002, the same research group [145] conducted another systematic review which concluded that there was still insufficient information to recommend surgery over conservative treatment, or vice versa. Lynch and Renstrom [130] commented that surgical treatment to ankle lateral complex may induce some serious, though infrequent complications. Functional conservative treatment was free of complications, and did not produce late symptoms than surgical repair and casting, therefore, there was a growing consensus to treat grade III sprains firstly with conservative functional treatment. If such treatment failed to enhance ankle function after a considerable period of time, surgical repair could be performed. Karlsson and Sancone [146] suggested that immobilization should never be used, not even in severe ankle sprain injury, as it may result in joint stiffness, muscle atrophy, and loss of proprioception.

For syndesmosis ligamentous sprains, the common treatment was to prescribe a walking boot for four to six weeks [147]. The walking boot provided resistance to avoid the distal tibia and fibula to separate apart – a motion that imposed stress on the interosseous tibiofibular ligament between the distal tibia and fibula. The prescription lasted for four to six weeks for the interosseous tibiafibular ligament to heal.

Lastly, traditional Chinese medicine methods, such as herbs, massage and acupuncture, were well applied in China or managing sports injuries. They were treated as a kind of effective alternative method, especially in treating ankle ligamentous sprain injury. The effect was already widely reported in the Chinese literature, and also in numerous studies in the English literature, on its analgesic effect to relieve pain [148], reduce swelling [149] and edema [150], restoring normal ankle function [151,152].

### Sequela of ankle ligamentous sprain

Injuries to the lateral ligament of the ankle often led to ankle instability [153]. Previous studies reported that 74% of the patients who suffered from an inversion ankle sprain injury had persisting symptoms 1.5–4 years after the injury [154], in which 10–30% of patients may have chronic symptoms such as persistent synovitis or tendinitis, ankle stiffness, swelling, pain, muscle weakness and frequent giving-way [130]. Yeung and colleagues [34] conducted an epidemiology study and reported that for ankles sprained 1–4 times, the major residual problem was pain (24–28%), while for ankle sprained five times or more, instability problems arose and became the major sequela (38%). Chronic ankle instability was commonly divided into mechanical ankle instability and functional ankle instability [85,155]. Mechanical ankle instability referred to abnormal laxity of the ligementous restraints, while functional ankle instability referred to normal ligamentous restraint but abnormal function with recurrent episodes of ankle giving-way [130]. Hubbard and Hertel [156] suggested that mechanical ankle instability may lead to functional ankle instability, however, Birmingham and coworkers [157] reported that functional ankle instability could exist in the absence of mechanical ankle instability.

Chronic ankle instability could be diagnosed by various methods, including imaging [158], arthroscopic diagnosis [159], and some functional scoring system such as the Foot and Ankle Disability Index [160], Ankle Activity Score [161], and the Cumberland Ankle Instability Tool [162]. It could lead to a delayed peroneal muscle reaction time [163], an inferior ankle kinesthesia and joint position sense [164], inferior proprioception and evertor strength [19]. In dynamic motions, it caused significant ankle biomechanics changes in gait [165-167], in single leg drop jump [168], in hopping [169], in cutting maneuver [170], and in figure-of-eight running and side hopping [171]. Beside chronic ankle instability, injuries to the lateral ligament of the ankle may also occasionally lead to ankle osteoarthritis [172], sinus tarsi syndrome and subtalar joint instability [173], and osteochondral defect at the talar dome [174].

### Prevention of sport-related ankle sprain injury

Garrick and Requa [175] were the first research group to attempt to prevent ankle sprain injury. They reported that high-top shoe and prophylactic ankle taping were effective in reducing the ankle sprain injury rate among a group of 2,562 basketball players during a one year study period. In 1987, van Mechelen, Hlobil and Kemper [176] proposed a "sequence of injury prevention" which described how sport injury-related studies came together to form the research framework. The first step was to identify the extent of the sports injury problem by epidemiology studies. The second step established the aetiology and mechanism of injuries and the third step designed and introduced preventive measures. Finally, the effectiveness of the preventive measures was assessed by repeating the original epidemiology study (step one). From that time, numerous studies had been conducted to evaluate different strategies for ankle sprain injury prevention. The strategies could be divided into prophylactic devices, functional training, technique training, change of game rules, and education. [177].

For prophylactic devices, most attempts were on taping, bracing, and orthosis. The similarity of these devices was to wrap the ankle joint from the foot segment to the shank segment. Some studies suggested that these devices provided a mechanical support to resist the ankle inversion moment [178,179], but some suggested that it instead improved the proprioception and joint position sense [180-182] and thus maintained a proper anatomical position during landing [68,183]. The effectiveness of these devices in reducing the ankle sprain injury rate was reported in numerous studies [70,184-186]. The role of shoe in ankle sprain prevention was less clear [186]. Barrett and Bilisko [187] suggested that high-top shoe limited extreme range of motion, reduced the external stress, and enhanced proprioception of the ankle joint, while Robbins, Waked and Rappel [182] argued that modern athletic footwear impaired proprioception. In a combination, Rovere and coworkers [188] suggested a low-top shoe with a laced ankle stabilizer was effective in reducing ankle sprain injury.

Most functional training protocols consisted of stability and postural control exercises [189]. For examples, wobble balance board or ankle disk were often used in stability training [190], and their effects was demonstrated in various studies [139,191,192]. Technique training was also prescribed by some research groups. For example, Stasinopoulos [193] devised a technical training program on take off and landing technique during attack and two man blocks for volleyball players which was effective in reducing ankle sprain occurrence. The aim was to coach the players to perform a quick and long final approach step and jump straight to avoid landing on the centre line under the net or on the feet of other players. Scase and coworkers [194] devised a program to coach a group of junior elite Australian football player with safe landing, falling, rolling, and recovery skills, to avoid the common injury mechanism which was to land badly. The program was evaluated to be successful in reducing the incidence of ankle sprain injury, especially landing-related injury.

Game rules also played significant contribute to the occurrence of injury. Andersen, Engebretsen and Bahr [195] reported that less than one-third of the injuries seen on video were called foul in a one-year prospective study on the Norwegian professional football league. They concluded that there may be a need for an improvement of the game rules to protect players from dangerous play. In volleyball, Reeser and coworkers [196] proposed a change of rule to make any centreline contact within the conflict zone between the attacker and blocker a fault, as they believed that the majority of ankle sprain injury in volleyball happened when the players collided near the net. In rugby, injuries were believed to have association with match speed and the impact forces during physical collision and tackles. Gabbett [197] introduced a limited interchange rule to the game, and found that the injury rate was significantly reduced in a one-year prospective study. It was suggested that the match speed and impact forces were reduced because the players got fatigued due to the limited interchange rule. Last but not the least, education to athletes was also important. In a three-day netball competition in New South Wales in Australia and 1995, Hume and Steele [198] reported that only 5.1% wore high-cut shoes even if this was advised before the competition. Furthermore, although players had been advised to seek immediate treatment when injured, 54.7% of players finished the game before seeking treatment. They suggested that compliance to advice from sports medicine specialists, together with the research into the effectiveness of different injury prevention strategies, were both important to the injury prevention and safety promotion in sports [199].

## Conclusion

This paper summarizes the current understanding on acute ankle ligamentous sprain injury, which is the most common single type of sport-related trauma. As there is a global trend of mass participation in recreational sports, treating and preventing ankle sprain injury will become essential topics in sports medicine. Inadequate management to ankle sprain injury may lead to various sequelae and problems like functional instability and osteoarthritis. This article presents the ankle anatomy as the fundamentals, which is essential for understanding the injury and the subsequent research and improvement in treatment and prevention. Anterior talofibular ligament (ATFL) was the weakest ligament in the lateral ankle was most often getting injured in ligamentous sprain injury. Risk factors, aetiology and mechanism of ankle sprain injury were presented from a summary of previous studies. Players with a history of ankle sprain, wearing shoes with air cells in the bell and players who did not stretch before exercising were 4.9, 4.3 and 2.6 times more likely to sprain their ankle. Foot positioning and the reaction time of the lateral peroneal muscles were identified as the aetiology of ankle sprain injury. While in ankle supination sprain, there was an ankle inversion plus internal twisting of the foot, plantarlfexion with the subtalar joint adducting and inverting, and sometimes external rotation of the lower leg. A 41–45 Nm external rotatory torque would cause ankle failure. For examining the injury, diagnostic techniques and grading systems are introduced. Ankle fractures were commonly diagnosed with radiography or the Ottawa Ankle Rules. Anterior drawer test and talar tilt test were commonly used to identify ligamentous injury, while Thompson test, tendonscopy and radio graphic assessment were used to examine tendon rupture. Different grading system classify different injury level by the severity of injury to the ligaments, combined clinical presentation, the instability of ankle, and some consider multiple discipline with a scoring scale. The management, sequela and some suggestions of preventive strategies to be implemented are introduced. RICE treatment was commonly applied on-field, while Cryotherapy, ice-cooling were used in the first week after injury. Several functional treatment, braces and traditional Chinese medicine methods were usually applied after five days of injury. This review allows the reader to catch up with the previous researches on ankle sprain injury, and facilitate the future research idea on sport-related ankle sprain injury.

## Competing interests

The authors declare that they have no competing interests.

## Authors' contributions

DTPF conducted the revise and drafted the manuscript. YYC and KMM assisted in compiling the data and summary. PSHY interpreted the findings and critically revised the manuscript. KMC conceived and coordinated the study. All authors read and approved the final manuscript.
